# Conceptual and practical challenges associated with understanding patient safety within community‐based mental health services

**DOI:** 10.1111/hex.13660

**Published:** 2022-11-12

**Authors:** Phoebe Averill, Charles Vincent, Gurpreet Reen, Claire Henderson, Nick Sevdalis

**Affiliations:** ^1^ Centre for Implementation Science, Health Service and Population Research Department, Institute of Psychiatry, Psychology and Neuroscience King's College London London UK; ^2^ Department of Experimental Psychology University of Oxford Oxford UK

**Keywords:** community mental health services, mental health, patient safety, quality of care

## Abstract

**Introduction:**

Patient safety problems stemming from healthcare delivery constitute a global public health concern and represent a pervasive barrier to improving care quality and clinical outcomes. However, evidence generation into safety in mental health care, particularly regarding community‐based mental health services, has long fallen behind that of physical health care, forming the focus of fewer research publications and developed largely in isolation from the wider improvement science discipline. We aimed to investigate the state of the field, along with key conceptual and empirical challenges to understanding patient safety in community‐based mental health care.

**Methods:**

A narrative review surveyed the literature to appraise the conceptual obstacles to advancing the science of patient safety in community‐based mental health services. Sources were identified through a combination of a systematic search strategy and targeted searches of theoretical and empirical evidence from the fields of mental health care, patient safety and improvement science.

**Results:**

Amongst available evidence, challenges in defining safety in the context of community mental health care, evaluating safety in long‐term care journeys and establishing what constitutes a ‘preventable’ safety problem, were identified. A dominant risk management approach to safety in mental health care, positioning service users as the origin of risk, has seemingly prevented a focus on proactive safety promotion, considering iatrogenic harm and latent system hazards.

**Conclusion:**

We propose a wider conceptualization of safety and discuss the next steps for the integration and mobilization of disparate sources of ‘safety intelligence’, to advance how safety is conceived and addressed within community mental health care.

**Patient and Public Contribution:**

This paper was part of a larger research project aimed at understanding and improving patient safety in community‐based mental health care. Although service users, carers and healthcare professionals were not involved as part of this narrative review, the views of these stakeholder groups were central to shaping the wider research project. For a qualitative interview and focus group study conducted alongside this review, interview topic guides were informed by this narrative analysis, designed jointly and piloted with a consultation group of service users and carers with experience of community‐based mental health services for working‐age adults, who advised on key questioning priorities.

## 1 INTRODUCTION

Physical healthcare services have benefited from over two decades of patient safety research, a discipline concerned with ‘the avoidance, prevention and amelioration of adverse outcomes or injuries stemming from the process of healthcare’.^1^ Despite achievements within other clinical specialities, safety in mental health care is largely uncharted. However, interest in the safety and quality of mental health care is gathering momentum. In the United Kingdom, recent publications have advanced learning about areas such as care transitions, service user, carer, and provider perspectives on safety, and factors affecting incident reporting.^2^, ^3^, ^4^, ^5^, ^6^, ^7^, ^8^ However, further efforts are required to unify research on safety in mental health care with research from the physical healthcare‐focused patient safety science tradition.^9^

Existing research into safety in mental health care has almost exclusively prioritized inpatient services. This reflects comparable challenges to those documented in physical healthcare literature, where measuring safety and implementing interventions is more complex in community settings due to a lack of robust safety indicators, lower frequency of care encounters and limited ability to influence treatment adherence or the safety of patient home environments.^10^, ^11^ Despite these commonalities, there are likely safety concerns unique to community mental healthcare settings. For example, issues surrounding risky behaviour, decision‐making capacity and compulsory treatment pose specific challenges.^12^, ^13^

### 1.1 Aims and overview

In this paper, we aim to provide a brief narrative review of the present state of patient safety with a focus on community‐based mental health services. We seek to illuminate key conceptual and empirical challenges associated with understanding safety in the aforementioned settings. Finally, considering research gaps and the limitations of existing safety knowledge, we propose an approach to consolidating and furthering the evidence base for safety in community mental health care.

As issues concerning general services for working‐age adults are better documented, we focus primarily on this population (rather than services for children, older adults or specialist community care pathways, e.g., mental health learning disabilities care). Herein, community‐based mental health services are defined broadly as noninstitutional mental health services which deliver care to people living in community settings. This includes provision within primary care (e.g., care from a general practitioner [GP] or Improving Access to Psychological Therapies services), and secondary care mental health services (e.g., Community Mental Health Teams). Multidisciplinary care in these settings may involve a range of clinical professionals, including mental health nurses, psychiatrists, social workers, clinical psychologists and occupational therapists. Likewise, given the paucity of research exploring patient safety issues in community‐based mental health care, in places, we draw upon literature from psychiatric inpatient settings to illustrate the issues discussed. In these cases, we reflect upon further challenges introduced when applying safety concepts to community mental health settings.

## 2 METHODS

### 2.1 Study design

We conducted a narrative review to critically assess extant research, appraise knowledge gaps and examine conceptual issues within the field, seeking to contribute to the conceptual advancement of patient safety science as applied to community‐based mental health care. The present research was devised in response to difficulties encountered in our efforts to formulate appropriate search and screening criteria for a separate systematic scoping review by the same authorship team, which focused on the nature of patient safety problems in these services. Issues comprised problems in identifying relevant articles, lack of consensus over safety‐relevant outcomes in community‐based mental health care and difficulties in establishing the boundaries of safety and harm preventability within these settings.

Given these unresolved challenges, a narrative review was deemed a necessary initial step to take stock of this research area. The benefits of narrative review approaches lie in their permitting of a broader exploration of topics,^14^ and contribution to the conceptual development of a given area. Historical narrative reviews have been described as ‘irreplaceable to track the development of a scientific principle or clinical concept’, where the ‘narrative thread could be lost in the restrictive rules of a systematic review’ (p. 231).^14^

### 2.2 Literature search and selection

This review drew upon literature identified through several means. A subset of articles was retrieved from a systematic search developed for the separate systematic scoping review discussed above.^15^ This search, executed in June 2020, focused around three key elements: ‘mental health’, ‘patient safety’ and ‘community‐based mental healthcare’. Once the need for the present narrative exploration was ascertained, further targeted searches were performed, centring on investigating identified challenges detailed within this paper. These searches focused on mental health care and the wider fields of patient safety and improvement science, also involving patient safety literature developed in other comparatively better‐evidenced care settings, such as general hospital services.

Articles were purposively selected for inclusion according to their conceptual contribution to the debates addressed within this review,^16^ and were discussed and agreed upon amongst the review team. This review sought to provide an overview of a broad range of issues, rather than comprehensive coverage of all relevant papers. A diversity of literature was surveyed, with no restrictions applied on the basis of study design or publication status (e.g., ‘grey’ literature). Whilst the study limitations, suitability of methods and quality of obtained findings were considered,^14^ no formal quality assessment was undertaken. Findings were synthesized narratively and organized around key conceptual themes. The review was devised according to guidelines for the quality assessment of narrative review articles.^17^

## 3 RESULTS AND DISCUSSION

This narrative analysis was informed by 71 sources, including empirical research and contextual material such as policy documents and healthcare news announcements. A combination of published (70.4%) and unpublished (29.6%) literature was purposively selected for inclusion, focusing on a range of different care settings, including community‐based mental health services (17.0%), inpatient mental health services (19.7%) or mixed mental healthcare settings (47.9%). Other included sources related to physical healthcare services (2.8%), or mixed healthcare settings (12.7%). Of the literature consisting of research or reviews of research, 30.0% reported on quantitative data, 21.7% on qualitative data and 31.7% had mixed study designs, with a further proportion constituting editorials or position pieces (16.7%).

### 3.1 The current state of the field

In their review of the UK National Health Service (NHS) mental healthcare provision, the Care Quality Commission cited safety as a key priority.^18^ Some 43% of community‐based mental health services for working‐age adults were rated ‘requires improvement’ for safety at the most recent inspection.^19^ Likewise, an independent investigation into the safety of risk assessment in community mental health teams was recently announced.^20^

#### 3.1.1 The developing evidence base for mental health patient safety

Mental health patient safety research has focused predominantly on hospital settings,^21^ which constitute a minority of mental healthcare encounters. Service users' care journeys are increasingly comprised of contact with community‐based mental health services, with UK psychiatric inpatient bed numbers falling by over 55% since 2000,^22^ and efforts to reduce the length of inpatient stays and avoid admissions through community‐based alternatives.^23^ Consequently, community services are treating larger numbers of sicker patients.^24^

Nevertheless, a handful of studies have included data about patient safety concerns across several types of mental healthcare settings, with participants reporting on experiences of inpatient or community services.^4^, ^5^, ^25^, ^26^ Some study findings are of evident relevance to community care, in highlighting safety risks relating to lengthy community treatment waiting times, care discontinuity, inadequate crisis services and challenges in managing acute risk in the community.^4^, ^5^, ^25^ Different perceptions of safety in the community versus hospital settings were also directly discussed in one paper.^4^ Other insights are likely of relevance across all mental healthcare settings, though may not have been explicitly explored or evidenced in terms of community services within the articles. These include risks stemming from workforce issues such as inadequate staffing levels, training and staff burnout.^4^, ^5^, ^25^ It is plausible that data have not always been disaggregated to analyse by mental healthcare setting type, which could form a pertinent point of analysis in future work. Indeed, a study of staff‐reported risk assessment and safety management processes revealed important differences in risk assessment practices between mental healthcare settings, including a greater focus on patients' family and social context in the community than in hospital‐based care.^26^ Further research centring explicitly on issues of safety in community‐based mental health services is however warranted.

Aside from gaps in the evidence base, the research field faces further challenges. There is a lack of integration between the field of safety in mental health care and the wider safety and quality evidence base.^9^, ^27^ Much of the research about safety in mental health care has neglected to acknowledge and build upon established patient safety science literature, developed primarily in physical healthcare contexts. Indeed, a substantial body of literature discusses topics widely regarded as safety‐relevant (e.g., self‐harm, violence and aggression), though does not necessarily situate itself within existing patient safety theory, such as human factors or systems approaches.

This divide is mirrored within the quality and safety academic discipline. In searches of three journals containing traditional patient safety literature, few titles corresponding to safety in mental health settings were identified.^9^ The authors determined that research into safety in mental health care tended instead to be published within mental health‐specific journals.^9^ This separation impedes system‐wide learning on safety events and principles which apply across clinical settings.

A further body of evidence discusses issues likely to affect safety across the care journey (e.g., care team communication problems), though neither embeds relevant patient safety science literature, nor is it conceived of as safety‐relevant research. Such articles instead identify themselves within the spectrum of care quality,^28^ or focus on specific issues, such as inadequate care planning,^29^ without linking these findings to safety implications. A bibliometric study of research activity on patient safety in community mental health services exemplifies this problem.^30^ Searches of ‘patient safety’ and ‘community mental health services’ across two bibliographic databases retrieved only two articles covering safety in community mental health care, neither of which centred specifically on this setting.^31^, ^32^ The apparent lack of relevant research is likely in part due to the failure of researchers to contextualize their findings within the parameters of patient safety.

#### 3.1.2 Dominant approaches to safety in mental health care

Service users' interests have not been placed at the heart of a nascent focus on improving safety in mental health care, where ‘safety’ has typically been approached as the inverse of risk in these services.^33^, ^34^, ^35^ Since widespread moves away from institutionalization towards community care, incidents of violence and aggression have received significant attention, due to high‐profile public inquiries and media coverage of a small number of homicides by service users.^36^, ^37^ This focus is further sustained by rates of patient assault on mental health staff, which are the highest amongst the healthcare sector.^12^, ^38^

The importance of staff safety cannot be overstated, and this issue may have interrelated consequences for patient safety. Workplace violence is associated with poor well‐being and burnout amongst healthcare staff, which in turn link to factors affecting patient safety and care quality.^39^, ^40^ For example, beyond potential safety consequences immediately following an assault, such as a risk of patient injury during physical restraint,^41^ repeated exposure to staff‐directed violence may contribute to workforce issues including absenteeism and staff turnover, and overuse of restrictive practices.^42^, ^43^ Such issues provoke further safety risks. Nevertheless, efforts to improve safety in mental health care must not be limited to violence reduction.

In prioritizing risks that service users may present to themselves or others when acutely unwell, the term ‘patient safety’ has been somewhat misappropriated. This interpretation situates patients as the origin of risk, prohibiting discussion about iatrogenic harm or hazards elicited by the process of health care itself.^27^, ^44^ The corresponding risk management culture has resulted in a narrow safety research agenda, centred around topics such as suicide, self‐harm and violence. Indeed, a recent review exploring patient involvement in the development of safety improvement interventions in acute inpatient mental healthcare settings identified that almost two thirds of included studies were concentrated on the reduction of restrictive practices, rather than on interventions aimed at advancing the therapeutic culture of these settings.^45^ Where resource allocation, team culture and training are concentrated primarily around maintaining personal and public safety,^35^, ^46^ there may be overreliance on coercive or restrictive interventions, curtailing a broader focus on proactive promotion of safe care.^33^ Possible sources of harm in community‐based mental health care are summarized in Box 1.

Box 1. **Potential areas for patient harm in community‐based mental health services**

**Table:** 

Harm from ineffective risk management	Harm from inadequate or unsuccessful prevention and management of risk, such as self‐harm, suicide or risks of violence and aggression. Capacity for prevention of these events by services may not always be clear.
Harm due to failure to provide appropriate treatment	Service users routinely do not receive optimal or evidence‐based standards of care, which may contribute to harm. For example, staffing shortages may result in service users not being assigned a care coordinator when one is required, or not receiving care within safe timeframes.
Medication‐related harm	Medications prescribed for mental health problems may result in adverse drug reactions, unpleasant or harmful side‐effects, or contribute to the development of comorbid physical health conditions. Medication errors, on part of care teams or service users and their carers, may also result in harm. This is increasingly relevant in community care, where service users play a larger role in their own medication management.
Harm from restrictive or coercive care	Harm may stem from the use of restrictive practices in mental health care, including scenarios where there is contact with other services which are less equipped to address a mental health crisis (e.g., the police). Service users may feel that they have little control over their own lives.
Harm due to undertreatment	Avoidable harm may result from under‐detection and undertreatment of risks associated with prescribed medications, such as failure to prescribe metformin for antipsychotic‐induced dyslipidaemia. Similarly, access to interventions, such as psychological therapies, amongst service users who would benefit from such treatment may be inequitable and more readily offered to those perceived to be assertive or articulate.
Harm relating to diagnosis	Misdiagnosis, missed diagnosis or delayed diagnosis can cause harm by delaying access to the appropriate course of treatment. Delays may contribute to deterioration and loss of confidence in mental health services. Service users may also experience harm associated with the specifics of the diagnosis they receive. For example, those with a personality disorder diagnosis may be faced with lack of adequate treatment pathways or stigma from care teams.
Psychological harm	Unhelpful or distressing encounters with community‐based mental health services may cause service users to feel unsafe when using these services. Similarly, prior experiences of compulsory treatment under Mental Health Act legislation may erode trust in care systems, potentially leading service users to conceal important risk information from care teams.

### 3.2 Key challenges for understanding patient safety in community mental health care

In what follows, we discuss theoretical and empirical issues in understanding and conceptualizing safety in community mental health care.

#### 3.2.1 Defining patient safety in mental health care

To date, patient safety definitions have been derived largely from physical healthcare contexts.^6^ Concepts of adverse events, errors and near misses are also shaped by terminology originating outside of mental healthcare settings.^21^ The United States Agency for Healthcare Research and Quality offer a well‐cited definition of patient safety as: ‘freedom from accidental or preventable injuries produced by medical care’.^47^

The focus on ‘medical care’ offers little scope to apply safety principles to the wealth of nonpharmacological treatments provided in mental health care, where the workforce itself has been described as the main therapeutic intervention.^48^ Additionally, positioning ‘injury’ as the safety outcome of interest obscures key types of iatrogenic harm. For instance, assessment and detention under Mental Health Act legislation can impact patients' psychological safety, potentially invoking trauma or replicating prior traumatic experiences.^49^ This example exposes further tensions for the concept of ‘patient safety’ in this care context. Community clinicians routinely face competing potentially harmful scenarios, whereby delaying or choosing not to pursue a Mental Health Act assessment on the grounds of providing the least restrictive level of care may increase risks of adverse safety outcomes associated with relapse in the community (e.g., suicide, harm to others).

The constraint of safety concerns to injuries ‘produced by’ healthcare services marks a further problem with this definition when applied to mental health care. As discussed, service users may be at risk from their own actions when acutely unwell (e.g., self‐harming behaviours). This definition may be poorly aligned with certain hazards prevalent in mental health care, but also exemplifies key disparities between mental health services compared to other care specialities. Indeed, mental health teams tend to focus on risks generated by service users, rather than by the care itself.^26^

Research also suggests that the poorly defined nature of patient safety in mental health care may create challenges for clinical care. Qualitative interviews of NHS psychiatrists revealed limited agreement on definitions of ‘patient safety’ and ‘quality’.^50^ Clinicians lacked awareness of the wider safety context in the NHS, including relevant high‐profile publications such as *An Organisation with a Memory*.^51^ Critically, participants failed to recognize certain types of potential safety incidents, culminating in shortfalls in both incident reporting and opportunities for learning.^50^ This is problematic, as whether a given factor is conceived of as a safety issue may impact awareness of these risks as they arise and motivation to take preventative measures.

#### 3.2.2 Conceptual complexities associated with safety in community settings

##### Locating the boundaries of the phenomenon of interest

Mental healthcare providers assume wider responsibility to care for the ‘whole person’, than do their physical healthcare counterparts. This is most pronounced within community services, where alongside mental healthcare provision, care teams have oversight of service users' needs in relation to housing, risk of victimization, financial problems and physical health, including comorbidities exacerbated by psychotropic medications.^52^, ^53^ When a service user comes to harm at home, it is difficult to disentangle the potential for prevention, if any, by mental health services. Equally, where a comprehensive harm definition is used, including subjective experiences of psychological harm associated with unfavourable care experiences, the line at which such events constitute a patient safety problem is ambiguous.

Mental health teams may also undertake clinical activities outside of their core expertise.^54^ Where a service user is reluctant to engage with their GP, psychiatrists and nursing staff have reported performing and interpreting physical healthcare investigations, such as phlebotomy and electrocardiograms, despite acknowledging their lack of confidence in carrying out these tasks.^53^ In these circumstances, one must consider whether imperfect test result interpretation is preferable to the absence of such investigations. These nuances must be reflected in our understanding of safety in mental healthcare contexts.

##### Measuring safety in long‐term care

Compared to acute psychiatric inpatient care, the longer‐term nature of patient journeys through community‐based services presents challenges for operationalizing safety. Efforts to understand safety in clinical practice have centred on so‐called active failures, corresponding incidents and their analysis.^1^ Severe incidents resulting in immediate, observable harm are most likely to be reported and selected for in‐depth clinical incident analysis. However, this may be at odds with the context of care delivery in community settings.

Safety problems which manifest in long‐term community care may be better understood by examining the dynamic accumulation of risk from unsafe care processes and care delivery problems over time. Unlike in hospital admissions, community‐based clinicians perform in the context of long‐term management of myriad complexities. For example, monitoring of stable or deteriorating chronic illnesses over time, rising multimorbidity and communication challenges from care delivery across multiple, fragmented settings are some of the difficulties teams face.^55^, ^56^, ^57^ Harm from such risks may be less evident, and less immediate, and their role in safety event causation may be less easily established. These complications thus align poorly with the traditional focus on specific errors, lapses and other ‘sharp end’ performance failures which are temporally or physically proximal to an incident and thus more easily measurable.

##### Community care is upstream and preventative

An overlapping challenge for observing and measuring safety concerns the upstream nature of much of community health care. Detection, prevention and maintenance of conditions are the mainstay of community‐based care, seeking to avert adverse outcomes such as deterioration or hospitalization. Consequently, the safety impacts of care in primary and community mental health care may not be quantifiable until further up the succession of health care, in terms of either causing or preventing adverse outcomes (e.g., psychiatric inpatient admissions, or long‐term psychotropic medication‐related physical health complications). Indeed, the consequences of misdiagnosis in primary care may not reach clinical attention for several years.^58^

Likewise, as clinical guidance is typically disorder‐specific,^58^ providers are sometimes obliged to depart from these decision‐making supports, to weigh up the relative benefits and potential harms of treatment. For example, clinicians may consider the cardiometabolic burden associated with long‐term antipsychotic medication, versus the potential reduction in suicide risk.^59^, ^60^ These examples reinforce the importance of attending to system‐wide care processes over time, rather than incidents alone.

### 3.3 Conceptualization of patient safety in a community mental health setting: A case example

When seeking to understand safety in the community, several factors warrant consideration. Care journeys may span months to decades, with a much lower intensity of care offered than in inpatient settings. Accordingly, the pace of care may be much slower, often with little to no community team involvement between appointments. Access issues are of greater significance to safety in community care, where waiting times often extend over several months for specialist psychological therapy, or to receive any care upon referral to secondary care community services.^61^, ^62^, ^63^ Rather than a direct relationship between care delivery failures and immediate safety consequences, safety problems in community settings may less resemble an ‘incident’. Risks may build over time where care is delivered across multiple, dispersed community settings, with patients and their carers playing a bigger role in patient safety, alongside involvement from several providers (e.g., GP practice, community mental health team, social care, community pharmacy).

A worked example (see Box 2) illustrates the operation of risks and their influence on organizational safety across a 1‐year period in a patient journey. It is informed by a systems perspective, according to the Yorkshire Contributory Factors Framework mental healthcare adaptation (YCFF‐MH).^25^ This hypothetical scenario is based on a recent announcement by a UK pharmaceutical company of intentions to discontinue the production of Priadel® brand lithium carbonate modified‐release tablets.^64^

Box 2. **A hypothetical case example of a safety event in community‐based mental health services**

**Table:** 

Safety event	Relapse of service user with bipolar disorder after a long period of stability, caused by sudden discontinuation of lithium carbonate medication.
Outcome(s)	Psychiatric hospitalization, severe depressive episode and minor accidental physical injuries due to risky behaviours in manic episode.
Description of contributory factors	Due to the COVID‐19 pandemic, guidance was issued recommending that lithium monitoring intervals were to be increased from 6 up to 9 months for stable patients (April 2020). Service user had been responding well to lithium carbonate (Priadel® brand) for 2 years whilst under the care of Community Mental Health Team. Six‐monthly routine physical monitoring indicated no abnormalities (blood levels within therapeutic range, no problems with renal or thyroid function). Due to stability on medication regimen, service user was discharged from secondary care for continued monitoring in primary care. Service user informed by care coordinator that their GP practice would contact them directly when required to arrange a follow‐up consultation in primary care (July 2020). No documentation of secondary care discharge letter in GP records. Therefore, Community Mental Health Team still presumed to be responsible for medication and physical health monitoring. No attempt made to contact service user (July 2020). Essential Pharma Ltd. announced a discontinuation of Priadel® from April 2021 onwards (September 2020). GP practice began to contact service users prescribed with Priadel® to arrange medication reviews and to plan transition to a different brand of lithium. Service users only contacted if records indicate that there is no current Community Mental Health Team care package in place. No check made on whether secondary care services were managing these transitions for their caseloads. No attempt made to contact service user (September 2020). Service user running low on their medication supply and was delayed in submitting repeat prescription to pharmacy (February 2021). Pharmacy supplies of Priadel® had already run out when service user submitted repeat prescription form (March 2021). Pharmacist explained to service user about discontinuation of Priadel® brand, recommended alerting their GP and requesting prescription review. Pharmacist failed to advise service user to contact NHS 111 for advice or emergency prescription. No check of how many tablets service user had left (March 2021). Service user abruptly ran out of medication before contact could be made with GP. Sudden discontinuation led to acute episode of mania resulting in hospitalization (March 2021).

Lithium carbonate is prescribed for mood stabilization in individuals with bipolar disorder and treatment‐resistant depression. Due to its narrow therapeutic index, regular monitoring must be undertaken, owing to the risks of toxicity. The Priadel® brand of lithium carbonate is widely used in the United Kingdom, with over 750,000 prescriptions of either the 200 or 400 mg strength tablets documented by GP practices across England in the year preceding November 2020.^65^ Due to pressure from prescribers,^66^ the Department for Health and Social Care reached an agreement with the manufacturers for continued supply of the medication to the United Kingdom.^67^

**Figure 1:**
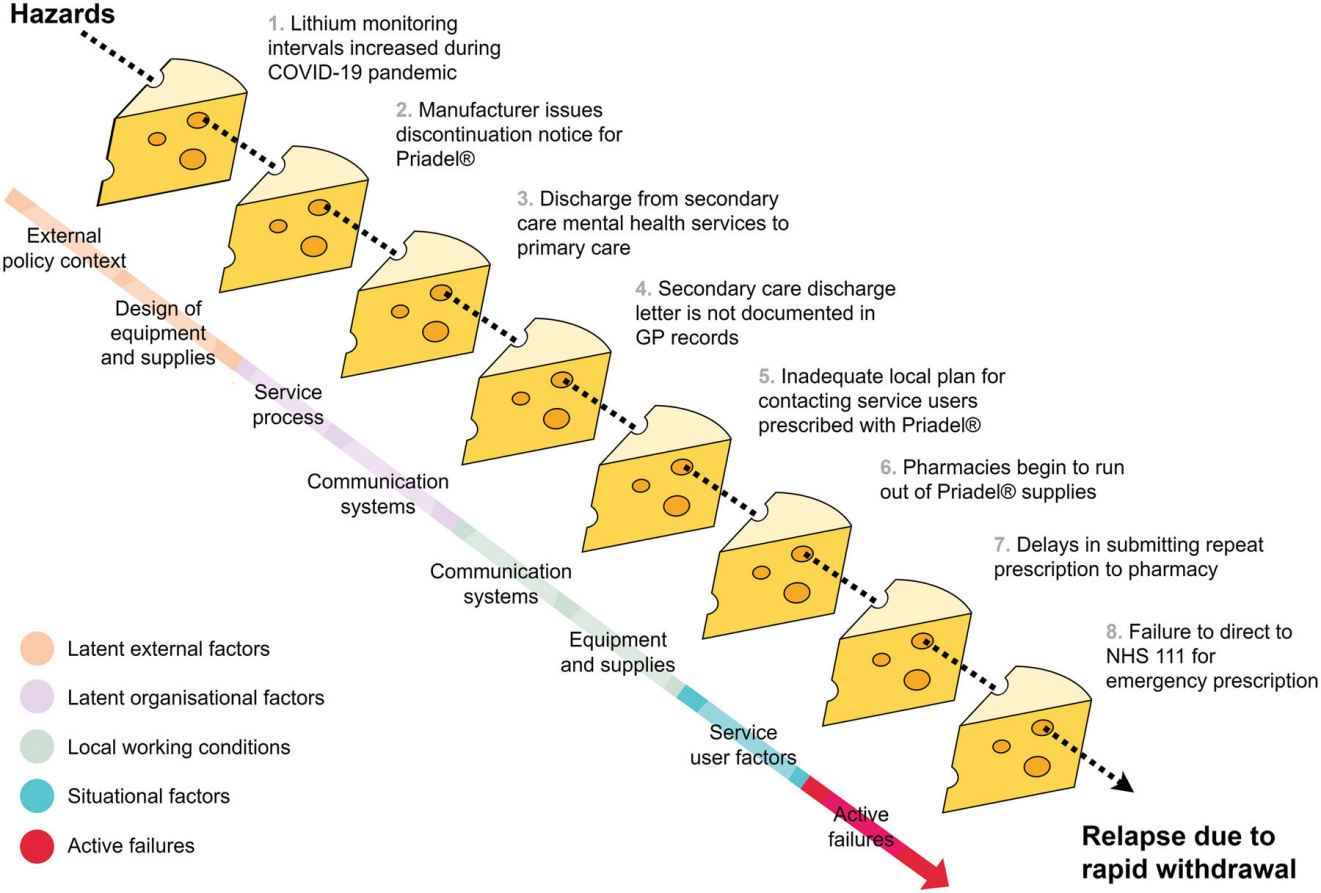
Swiss Cheese Model of an adverse event in community‐based mental health care mapped to the Yorkshire Contributory Factors Framework—Mental Health (adapted from Berzins et al.^25^ and Reason^76^).

This scenario, based on its hypothetical discontinuation, exemplifies the importance of investigating safety over a longer period of patients' community care journeys. Event reviews confined to so‐called ‘sharp end’ factors immediately preceding the service user's hospitalization would likely fail to capture broader systemic problems, obscuring key sources of learning. The roles of multiple contributory factors across the wider system are exhibited, including changes to national care guidance, communication problems and active failures. These accumulating hazards, which successfully surmount defences built into the system, are depicted across the trajectory of a patient's care journey in Figure 1.

### 3.4 Implications and proposed directions for future research and practice

Seeking a resolution to the problems described, in what follows, we propose several directions for safety science applied to community mental health care (Box 3).

Box 3. **Looking forward: Next steps to advance mental health patient safety in the community**

**Table:** 

Agreed definitions of safety	Shared definitions of what constitutes a patient safety problem in this context must be developed, to agree on an agenda for improving safety.
A wider remit for safety	Evaluation of ‘safe’ community services must not be centred around a limited number of recognized adverse incidents only (e.g., incidents of violence and aggression). Attention must be paid to what makes service users feel safe or unsafe, along with broader, upstream determinants of safety (e.g., safe waiting times).
Measure safety over time	Efforts to investigate and measure safety in community‐based mental health care must be designed with long‐term care journeys in mind.
Make greater use of theory and evidence	Opportunities should be identified to learn from existing patient safety theory and evidence, which may have its origins within other care specialties or settings.
A nuanced approach to intervention	A wider range of safety interventions are needed to target systems factors which impact the safety of care, moving beyond a focus on direct service user and staff factors alone.
Draw on wider sources of ‘safety intelligence’	Beyond traditional academic evidence, we must also seek to understand what can be learned from existing unpublished literature and local quality improvement work. Likewise, our approach to safety improvement in community services must be shaped by and with mental health service users and carers.

#### 3.4.1 A need for shared definitions of patient safety in mental health care

Without shared definitions, nomenclature and agreed indicators of safety as applied to mental health care, research has observed safety through an overly narrow lens. A significant proportion of published research in this field concerns self‐harm and suicides among mental health patients, due to the severity of harm caused by these incidents.^68^, ^69^, ^70^ Although this research is vital to suicide reduction, other key risks must be identified and targeted in future research.

Efforts are required to define and determine the remit of patient safety in mental health care, which influences the aims of corresponding interventional work. It has been argued that safety in mental health care can be defined narrowly or broadly, with the former comprising a series of adverse events such as suicide and medication errors and the latter encompassing wider matters pertaining to the quality of care, service access and stigma.^21^ An ambitious, broader mandate risks weakening the impact of efforts to improve safety, yet the authors anticipate that an overly narrow conceptualization could undermine its effectiveness, by failing to target underlying systemic factors which obstruct safe care.^21^ Standardization of language, definitions and development of practice standards in mental health patient safety are important prerequisites to effective safety measurement and improvement.^12^

#### 3.4.2 A broader conceptualization of safety

Given the unmapped nature of research into safety in community‐based mental health services, a robust approach to conceptualizing safety problems and wider system‐level risk factors is essential. The following safety definition provides a starting point by beginning to accommodate the specifics of mental health care: ‘the avoidance of unintended unsafe or iatrogenic harm associated with mental healthcare—either an error in inappropriate treatment or an omission to detect unsafe behaviour’.^71^ However, terminology directly associated with ‘sharp end’ failures or incidents (e.g., ‘error’, ‘omission’), may not adequately represent latent, systemic contributors to safety that better reflect community care processes. This definition must be expanded to encompass the full range of safety issues associated with community care.

The appeal for a broader safety agenda in mental health care is echoed widely. Creative research methods are thought necessary to overcome dominant views of patient safety in mental health care,^72^ which may have ‘filtered out’ certain safety considerations, such as the use of practices which cause patients to feel distress and powerlessness. Other researchers note that the boundaries of safety are blurred in community mental health care, aligning more closely with what has been conceptualized as ‘quality’ issues in other specialities.^73^ It is plausible that accumulated poor‐quality care experiences may culminate in less safe care.

#### 3.4.3 Evaluate safety across the whole care journey

Recognizing the complexity of measuring safety in long‐term and community care scenarios, a departure is needed from a simplistic incident‐focused perspective, to suitably capture key determinants of safety which impact care over time (Figure 1). As such, a wider frame of safety event analysis is required to incorporate the whole patient journey.^74^ Regarding community care, it has been asserted that: ‘The concept of a patient safety incident, or even of adverse events, breaks down in these settings or is at least stretched to its limit’ (p. 6).^57^ These considerations are pertinent to understanding safety in any long‐term, community‐based care scenario.

#### 3.4.4 Making greater use of safety theory and evidence

Without employing established patient safety theories and models, mental health services are unlikely to observe safety improvements beyond those already achieved by the immediate, service user and staff‐directed interventions. Incidents of violence or self‐harm on inpatient units have often been attributed to proximal factors,^75^ including attentional lapses by staff members, or patients labelled as ‘challenging’. However, the contributory role of wider organizational characteristics has seldom been explored.

A systems approach to safety views incidents in the context of dynamic, complex healthcare system factors which precipitate safety events.^76^ The role of latent conditions (e.g., organizational culture, service resourcing), alongside localized workplace factors (e.g., team characteristics, staffing levels), have been identified in multiple models delineating systems safety.^25^, ^76^, ^77^, ^78^ To advance the field, system‐wide conditions must be acknowledged.

#### 3.4.5 Developing a wider range of safety interventions

Interventions to improve safety in mental health care have primarily targeted patient and staff‐level factors only, with limited consideration of wider systems influences. For example, service users might receive directly therapeutic psychological interventions in response to self‐harming incidents. Likewise, care teams may receive training aimed at preventing errors or other performance‐related factors deemed relevant to the incident causation.

Whilst direct interventions are undoubtedly important, a broader, more nuanced approach is likely required to drive additive safety improvements. For instance, boredom due to inadequate activity provision in inpatient environments has been linked to aggression and self‐harming.^79^ Provision of structured evening activities (e.g., drama, animal therapy), was associated with reduced proportions of adolescent patients self‐harming during evenings.^80^

Similarly, the Safewards model,^81^ evidenced reductions in safety outcomes such as self‐harm, violence and restrictive practices within the inpatient environment, using an approach which addressed both local and systemic factors. Moreover, before‐and‐after analyses indicated that organizational and indirect factors (e.g., low staff turnover and family involvement in learning from suicides) were associated with reductions in suicide rates amongst mental health service users.^82^ These factors comprise part of a toolkit for specialist mental health services and primary care, aimed at improving safety.^83^ Together, these works suggest the causal interplay of both local and distal systemic conditions in safety events. For research to be best positioned to improve care, such factors must inform our understanding of organizational safety.

#### 3.4.6 A place at the table for a wider range of informants on safety

Going forward, echoing views expressed by other researchers, we call for closer collaboration between mental health care and the wider field of patient safety.^9^ Future mental health safety‐relevant research must position itself within the patient safety science discipline so that these services are acknowledged as a priority in global safety improvement agendas. Where synergies exist, safety research across different clinical specialities must aspire to build upon each other, rather than progressing in isolation. Moreover, for greater unification of the evidence base, barriers faced by quality improvement professionals to publishing their findings in traditional patient safety journals must be surmounted.^84^ Where there is mission alignment to improve safety, ‘messy’ real‐world improvement work of relevance to long‐term conditions must be represented alongside traditional research methodologies within our growing understanding of safety.

**Figure 2:**
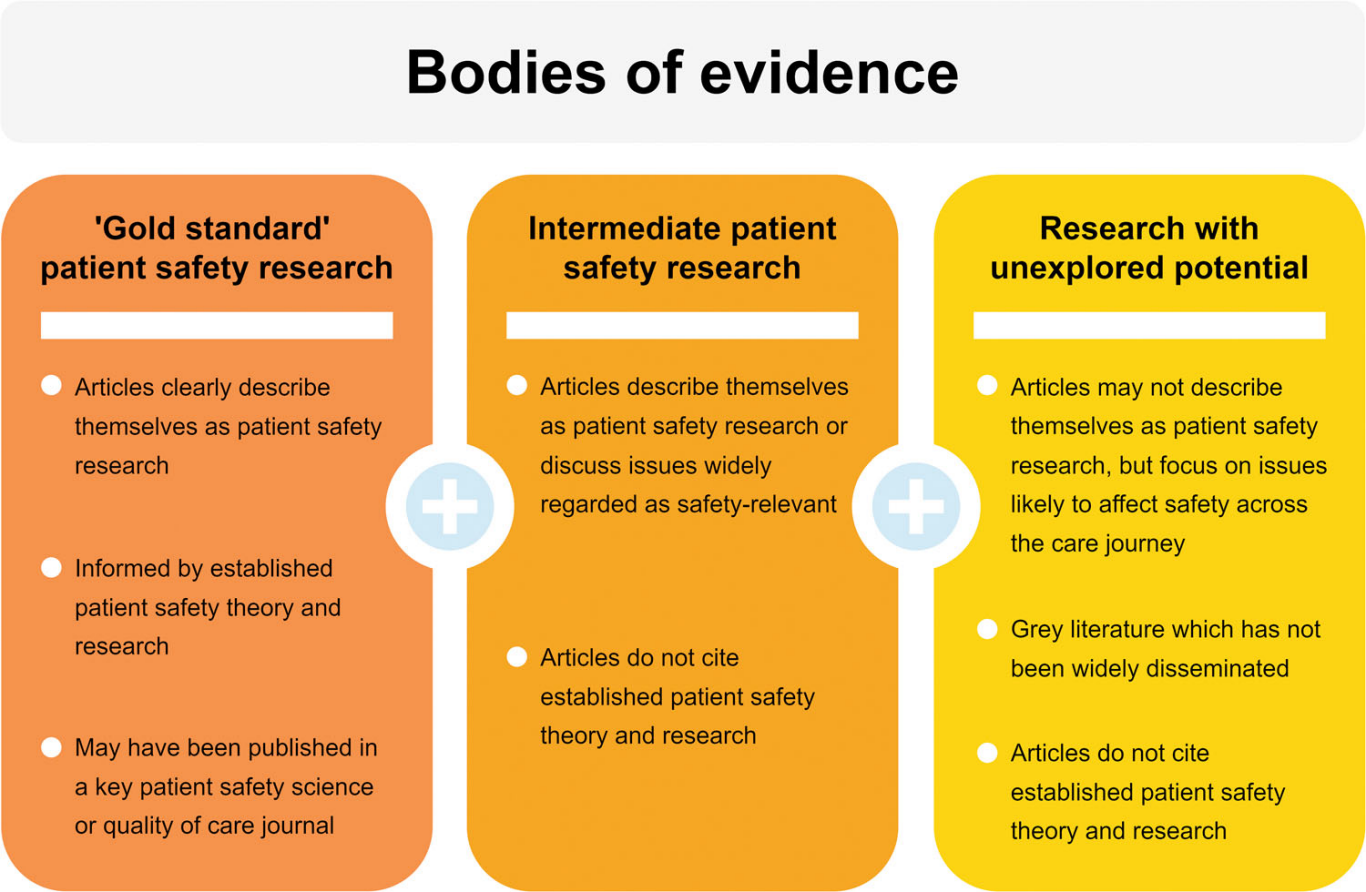
The integration of disparate bodies of evidence pertaining to mental health patient safety

However, to make the best use of existing evidence, our perspective of safety must not be limited only to research which fits well within both mental health and patient safety science research areas. This may obscure learning from other evidence bodies. Patient safety researchers must accommodate broader bodies of research, which may not yet have harnessed theories and principles from patient safety science, or have contextualized itself within the patient safety evidence base, alongside local service improvement reports or other ‘grey’ literature (see Figure 2).

Likewise, we support calls for a bottom‐up approach to safety in mental health care, both in terms of its conceptualization^85^ and improvement initiatives, with the involvement of service users, carers and frontline staff.^8^, ^45^ Historically, safety efforts may have been driven by top‐down policy and regulations, to the detriment of aims to produce authentic improvements to service users' care.^86^ In integrating diverse sources of safety intelligence, we will be best placed to improve care, by bringing together findings which are embedded in patient safety theory alongside those grounded in the reality of practice. This will provide a meaningful starting point for understanding safety in community mental health services.

## 4 CONCLUSION

Community‐based mental health services must be at the forefront of future endeavours to define, measure and intervene to improve patient safety in mental health care. Although recent increases in research into safety in mental health care are encouraging, future research programmes must seek to expand the evidence base beyond psychiatric inpatient settings. It is also essential that research and innovation are not constrained to a limited range of safety problems, such as suicide and self‐harm. Moreover, there is a lack of shared language and agreement over what constitutes a safety concern in the context of community‐based mental health services. Going forward, we argue that the disparate bodies of existing research, with unexplored potential for understanding safety, must be integrated into our developing understanding of patient safety. Likewise, service users and carers must be involved in efforts to improve the safety of services. We hope this exploratory review of theoretical, conceptual and empirical challenges and discussion of potential approaches to their resolution will be useful to those seeking to advance this area of research.

## AUTHOR CONTRIBUTIONS

All authors were involved in the conceptualization of this paper. Phoebe Averill developed the first draft of the manuscript. All other authors provided feedback on drafts of this paper, which were used to critically revise the manuscript. All authors have read and agreed to the published version of the manuscript.

## CONFLICT OF INTEREST

Nick Sevdalis is the director of London Safety and Training Solutions Ltd., which offers training in patient safety, implementation solutions and human factors to healthcare organizations. The remaining authors declare no conflict of interest.

## Data Availability

Data sharing is not applicable to this article as no new data were generated or analysed during the current study.
